# 2-[3-(4-Methoxyphen­yl)-1-phenyl-1*H*-pyrazol-5-yl]phenol

**DOI:** 10.1107/S1600536809004012

**Published:** 2009-02-25

**Authors:** Rukhsana Kausar, Amir Badshah, Muhammad Zia ul Haq, Aurangzeb Hasan, Michael Bolte

**Affiliations:** a Allama Iqbal Open University, Islamabad, Pakistan; b Department of Chemistry, Quaid-i-Azam University, Islamabad 45320, Pakistan; c Institut für Anorganische Chemie, J. W. Goethe-Universität, Max-von-Laue-Strasse 7, 60438 Frankfurt/Main, Germany

## Abstract

The title compound, C_22_H_18_N_2_O_2_, was derived from 1-(2-hydroxy­phen­yl)-3-(4-methoxy­phen­yl)propane-1,3-dione. The central pyrazole ring forms dihedral angles of 16.83 (5), 48.97 (4) and 51.68 (4)°, respectively, with the methoxy­phenyl, phenyl and hydroxy­phenyl rings. The crystal packing is stabilized by O—H⋯N hydrogen bonding.

## Related literature

For general synthesis, see: Ahmad *et al.* (1997 ▶). For synthetic applications, see: Beeam *et al.* (1984 ▶); Bonati (1980 ▶); Elguero (1983 ▶); Freyer & Radeglia (1981 ▶); Trofinenko (1972 ▶).
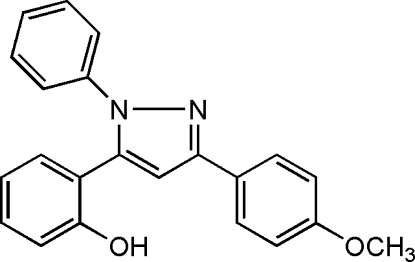


## Experimental

### 

#### Crystal data

C_22_H_18_N_2_O_2_
*M* *_r_* = 342.38
Monoclinic, 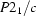

*a* = 9.5880 (5) Å
*b* = 13.7397 (8) Å
*c* = 14.2771 (7) Å
β = 109.340 (4)°
*V* = 1774.68 (16) Å^3^
*Z* = 4
Mo *K*α radiation
μ = 0.08 mm^−1^
*T* = 173 (2) K
0.33 × 0.31 × 0.26 mm

#### Data collection

Stoe IPDS-II two-circle diffractometer
Absorption correction: none
24784 measured reflections
4085 independent reflections
3676 reflections with *I* > 2σ(*I*)
*R*_int_ = 0.051

#### Refinement

*R*[*F* ^2^ > 2σ(*F* ^2^)] = 0.037
*wR*(*F* ^2^) = 0.096
*S* = 1.02
4085 reflections
241 parameters
H atoms treated by a mixture of independent and constrained refinement
Δρ_max_ = 0.28 e Å^−3^
Δρ_min_ = −0.21 e Å^−3^

### 

Data collection: *X-AREA* (Stoe & Cie, 2001 ▶); cell refinement: *X-AREA*; data reduction: *X-AREA*; program(s) used to solve structure: *SHELXS97* (Sheldrick, 2008 ▶); program(s) used to refine structure: *SHELXL97* (Sheldrick, 2008 ▶); molecular graphics: *XP* in *SHELXTL-Plus* (Sheldrick, 2008 ▶); software used to prepare material for publication: *SHELXL97*.

## Supplementary Material

Crystal structure: contains datablocks I, global. DOI: 10.1107/S1600536809004012/pk2149sup1.cif

Structure factors: contains datablocks I. DOI: 10.1107/S1600536809004012/pk2149Isup2.hkl

Additional supplementary materials:  crystallographic information; 3D view; checkCIF report

## Figures and Tables

**Table 1:** Hydrogen-bond geometry (Å, °)

*D*—H⋯*A*	*D*—H	H⋯*A*	*D*⋯*A*	*D*—H⋯*A*
O2—H2⋯N2^i^	0.91 (2)	1.88 (2)	2.7894 (11)	175.0 (17)

Symmetry code: (i) 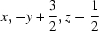.

**Fig. 1.**
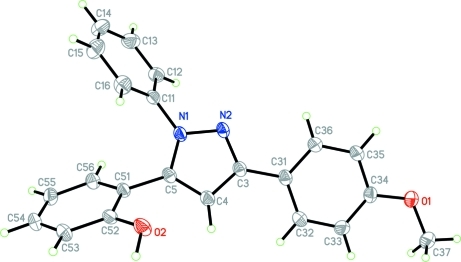
Molecular structure of the title compound with displacement ellipsoids at the 50% probability level.

**Fig. 2.**
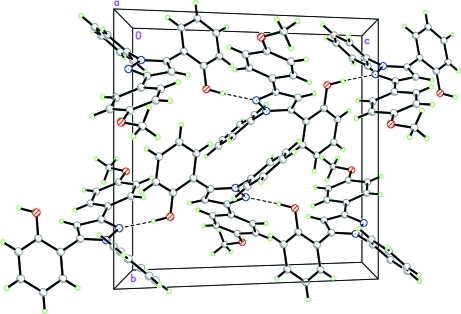
Packing diagram of the title compound.

**Fig. 3.**
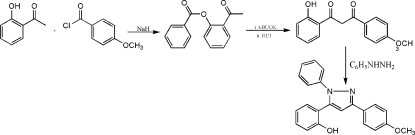
The formation of the title compound.

## supplementary crystallographic information

### Comment

Pyrazoles are important because of their potential for biological activity. They have antipruritic, anti-inflammatory and antirheumatic effects (Beeam *et al.*, 1984).  Both traditional and new scientific methods have been used to prepare new materials for medicine (Elguero *et al.*, 1983) and agriculture (Trofinenko, 1972). Neutral and anionic pyrazoles are excellent ligands and their coordination chemistry has been extensively studied (Bonati, 1980).  Pyrazoles are also used as analytical reagents (Freyer & Radeglia, 1981).  In the molecular structure of the title compound, C_22_H_18_N_2_O_2_, (Scheme 1, Fig. 1) the central pyrazole ring forms dihedral angles of 16.83 (5)°, 48.97 (4)° and 51.68 (4)° with the methoxyphenyl, phenyl and hydroxyphenyl rings, respectively. The crystal packing is stabilized by an O-H···N hydrogen bond (Table 1).

### Experimental

1-(2'-Hydroxyphenyl)-3-(4"-methoxyphenyl) propane-1,3-dione was prepared by a modified Baker Venkataram rearrangement as reported earlier (Ahmad *et al.* 1997). 2-(3-(4-methxyphenyl)-1-Phenyl-1*H*-pyrazol-5-yl)phenol was prepared by refluxing 1-(2'-hydroxyphenyl)-3-(4"-methoxyphenyl) propane-1,3-dione (2.7 g, 10 mmol) with phenyl hydrazine (1.08 g,0.99 ml, 10 mmol) in a mixture of 50 ml absolute ethanol and 15 ml glacial acetic acid for seven hours as shown in scheme 2.The oily mixture obtained was purified by a dry silica gel column. The product was recrystallized using absolute ethanol. (Yield: 45%, m.p: 449k)

### Refinement

All H atoms could be located by difference Fourier synthesis. They were refined with fixed individual displacement parameters [U(H) = 1.2 *U*_eq_(C) or U(H) = 1.5 *U*_eq_(C_methyl_)] using a riding model with C—H(aromatic) = 0.95Å or *C*—*H*(methyl) = 0.98 Å, respectively. The hydroxyl H atom was freely refined.

### Crystal data

**Table:**

C_22_H_18_N_2_O_2_	*F*(000) = 720
*M**_r_* = 342.38	*D*_x_ = 1.281 Mg m^−^^3^
Monoclinic, *P*2_1_/*c*	Melting point: 449 K
Hall symbol: -P 2ybc	Mo *K*α radiation, λ = 0.71073 Å
*a* = 9.5880 (5) Å	Cell parameters from 22780 reflections
*b* = 13.7397 (8) Å	θ = 3.8–27.6°
*c* = 14.2771 (7) Å	µ = 0.08 mm^−^^1^
β = 109.340 (4)°	*T* = 173 K
*V* = 1774.68 (16) Å^3^	Block, colourless
*Z* = 4	0.33 × 0.31 × 0.26 mm

### Data collection

**Table:**

Stoe IPDS-II two-circle diffractometer	3676 reflections with *I* > 2σ(*I*)
Radiation source: fine-focus sealed tube	*R*_int_ = 0.051
graphite	θ_max_ = 27.7°, θ_min_ = 3.7°
ω scans	*h* = −12→12
24784 measured reflections	*k* = −17→17
4085 independent reflections	*l* = −18→18

### Refinement

**Table:**

Refinement on *F*^2^	Secondary atom site location: difference Fourier map
Least-squares matrix: full	Hydrogen site location: inferred from neighbouring sites
*R*[*F*^2^ > 2σ(*F*^2^)] = 0.037	H atoms treated by a mixture of independent and constrained refinement
*wR*(*F*^2^) = 0.096	*w* = 1/[σ^2^(*F*_o_^2^) + (0.0463*P*)^2^ + 0.5301*P*] where *P* = (*F*_o_^2^ + 2*F*_c_^2^)/3
*S* = 1.02	(Δ/σ)_max_ < 0.001
4085 reflections	Δρ_max_ = 0.28 e Å^−^^3^
241 parameters	Δρ_min_ = −0.21 e Å^−^^3^
0 restraints	Extinction correction: *SHELXL97* (Sheldrick, 2008), Fc^*^=kFc[1+0.001xFc^2^λ^3^/sin(2θ)]^-1/4^
Primary atom site location: structure-invariant direct methods	Extinction coefficient: 0.030 (2)

### Special details

**Table:**

Geometry. All e.s.d.'s (except the e.s.d in the dihedral angle between two l.s. planes) are estimated using the full covariance matrix. The cell e.s.d.'s are taken into account individually in the estimation of e.s.d.'s in distances, angles and torsion angles; correlations between e.s.d.'s in cell parameters are only used when they are defined by crystal symmetry. An approximate (isotropic) treatment of cell e.s.d.'s is used for estimating e.s.d.'s involving l.s. planes.
Refinement. Refinement of *F*^2^ against ALL reflections. The weighted *R*-factor *wR* and goodness of fit *S* are based on *F*^2^, conventional *R*-factors *R* are based on *F*, with *F* set to zero for negative *F*^2^. The threshold expression of *F*^2^ > σ(*F*^2^) is used only for calculating *R*-factors(gt) *etc*. and is not relevant to the choice of reflections for refinement. *R*-factors based on *F*^2^ are statistically about twice as large as those based on *F*, and *R*- factors based on ALL data will be even larger.

### Fractional atomic coordinates and isotropic or equivalent isotropic displacement parameters (Å^2^)

**Table:**

	*x*	*y*	*z*	*U*_iso_*/*U*_eq_	
O1	−0.16266 (8)	0.90595 (6)	0.46141 (6)	0.0317 (2)	
O2	0.53923 (9)	0.76222 (6)	0.18033 (6)	0.02732 (18)	
H2	0.513 (2)	0.7794 (13)	0.1149 (15)	0.058 (5)*	
N1	0.54331 (9)	0.65343 (6)	0.43688 (6)	0.02103 (18)	
N2	0.44634 (9)	0.69329 (6)	0.47836 (6)	0.02183 (18)	
C3	0.33084 (11)	0.72394 (7)	0.40111 (7)	0.0216 (2)	
C4	0.35458 (11)	0.70369 (7)	0.31053 (8)	0.0233 (2)	
H4	0.2901	0.7185	0.2456	0.028*	
C5	0.49012 (11)	0.65812 (7)	0.33524 (7)	0.0209 (2)	
C11	0.68123 (11)	0.61521 (7)	0.50071 (7)	0.0208 (2)	
C12	0.68218 (12)	0.55147 (8)	0.57656 (8)	0.0256 (2)	
H12	0.5924	0.5341	0.5868	0.031*	
C13	0.81636 (13)	0.51336 (9)	0.63747 (9)	0.0335 (3)	
H13	0.8182	0.4697	0.6895	0.040*	
C14	0.94712 (13)	0.53896 (10)	0.62233 (10)	0.0402 (3)	
H14	1.0382	0.5123	0.6635	0.048*	
C15	0.94514 (13)	0.60346 (11)	0.54719 (11)	0.0408 (3)	
H15	1.0351	0.6213	0.5376	0.049*	
C16	0.81212 (12)	0.64223 (9)	0.48577 (9)	0.0302 (2)	
H16	0.8108	0.6865	0.4344	0.036*	
C31	0.20092 (11)	0.77139 (7)	0.41556 (8)	0.0221 (2)	
C32	0.06812 (12)	0.77997 (8)	0.33677 (8)	0.0268 (2)	
H32	0.0621	0.7544	0.2737	0.032*	
C33	−0.05622 (12)	0.82502 (8)	0.34791 (8)	0.0268 (2)	
H33	−0.1452	0.8301	0.2931	0.032*	
C34	−0.04804 (11)	0.86244 (8)	0.44031 (8)	0.0241 (2)	
C35	0.08451 (12)	0.85507 (9)	0.51998 (8)	0.0295 (2)	
H35	0.0905	0.8808	0.5829	0.035*	
C36	0.20682 (12)	0.81060 (9)	0.50782 (8)	0.0282 (2)	
H36	0.2960	0.8065	0.5625	0.034*	
C37	−0.29807 (12)	0.92114 (10)	0.38164 (9)	0.0363 (3)	
H37A	−0.2786	0.9593	0.3293	0.054*	
H37B	−0.3677	0.9565	0.4063	0.054*	
H37C	−0.3406	0.8581	0.3547	0.054*	
C51	0.56695 (10)	0.61401 (7)	0.27101 (7)	0.0205 (2)	
C52	0.58662 (11)	0.66787 (7)	0.19256 (7)	0.0216 (2)	
C53	0.65634 (12)	0.62480 (9)	0.13073 (8)	0.0287 (2)	
H53	0.6725	0.6618	0.0791	0.034*	
C54	0.70182 (12)	0.52839 (9)	0.14464 (8)	0.0306 (2)	
H54	0.7480	0.4995	0.1020	0.037*	
C55	0.68004 (12)	0.47382 (8)	0.22094 (8)	0.0279 (2)	
H55	0.7097	0.4075	0.2297	0.033*	
C56	0.61481 (11)	0.51682 (8)	0.28402 (8)	0.0244 (2)	
H56	0.6023	0.4799	0.3369	0.029*	

### Atomic displacement parameters (Å^2^)

**Table:**

	*U*^11^	*U*^22^	*U*^33^	*U*^12^	*U*^13^	*U*^23^
O1	0.0251 (4)	0.0429 (5)	0.0281 (4)	0.0094 (3)	0.0101 (3)	−0.0034 (3)
O2	0.0397 (4)	0.0253 (4)	0.0199 (4)	0.0030 (3)	0.0138 (3)	0.0021 (3)
N1	0.0234 (4)	0.0236 (4)	0.0191 (4)	0.0017 (3)	0.0110 (3)	−0.0009 (3)
N2	0.0247 (4)	0.0239 (4)	0.0207 (4)	0.0018 (3)	0.0126 (3)	−0.0017 (3)
C3	0.0256 (5)	0.0209 (5)	0.0209 (5)	0.0004 (4)	0.0111 (4)	0.0002 (4)
C4	0.0274 (5)	0.0248 (5)	0.0199 (5)	0.0028 (4)	0.0110 (4)	0.0016 (4)
C5	0.0262 (5)	0.0203 (4)	0.0190 (5)	−0.0006 (4)	0.0112 (4)	0.0004 (4)
C11	0.0216 (5)	0.0211 (4)	0.0203 (5)	−0.0012 (4)	0.0079 (4)	−0.0038 (4)
C12	0.0268 (5)	0.0274 (5)	0.0228 (5)	−0.0031 (4)	0.0086 (4)	−0.0004 (4)
C13	0.0376 (6)	0.0311 (6)	0.0267 (5)	0.0028 (5)	0.0039 (5)	0.0009 (5)
C14	0.0266 (6)	0.0460 (7)	0.0402 (7)	0.0074 (5)	0.0007 (5)	−0.0048 (6)
C15	0.0217 (5)	0.0527 (8)	0.0487 (7)	−0.0039 (5)	0.0127 (5)	−0.0075 (6)
C16	0.0283 (5)	0.0327 (6)	0.0332 (6)	−0.0063 (4)	0.0148 (4)	−0.0012 (5)
C31	0.0255 (5)	0.0217 (5)	0.0222 (5)	0.0015 (4)	0.0119 (4)	0.0007 (4)
C32	0.0296 (5)	0.0321 (6)	0.0208 (5)	0.0031 (4)	0.0110 (4)	−0.0012 (4)
C33	0.0251 (5)	0.0328 (6)	0.0223 (5)	0.0035 (4)	0.0077 (4)	0.0019 (4)
C34	0.0235 (5)	0.0246 (5)	0.0269 (5)	0.0029 (4)	0.0119 (4)	0.0007 (4)
C35	0.0282 (5)	0.0378 (6)	0.0231 (5)	0.0043 (4)	0.0095 (4)	−0.0070 (4)
C36	0.0258 (5)	0.0362 (6)	0.0224 (5)	0.0047 (4)	0.0075 (4)	−0.0037 (4)
C37	0.0255 (5)	0.0483 (7)	0.0336 (6)	0.0103 (5)	0.0078 (5)	−0.0015 (5)
C51	0.0206 (4)	0.0241 (5)	0.0183 (4)	−0.0010 (4)	0.0086 (4)	−0.0025 (4)
C52	0.0223 (4)	0.0254 (5)	0.0175 (4)	−0.0009 (4)	0.0071 (4)	−0.0019 (4)
C53	0.0318 (5)	0.0375 (6)	0.0212 (5)	0.0030 (5)	0.0144 (4)	0.0007 (4)
C54	0.0303 (5)	0.0399 (6)	0.0253 (5)	0.0061 (5)	0.0143 (4)	−0.0055 (5)
C55	0.0274 (5)	0.0271 (5)	0.0304 (5)	0.0039 (4)	0.0113 (4)	−0.0045 (4)
C56	0.0258 (5)	0.0247 (5)	0.0249 (5)	−0.0001 (4)	0.0115 (4)	−0.0003 (4)

### Geometric parameters (Å, °)

**Table:**

O1—C34	1.3694 (12)	C31—C32	1.3971 (15)
O1—C37	1.4307 (14)	C31—C36	1.4067 (14)
O2—C52	1.3657 (13)	C32—C33	1.3984 (15)
O2—H2	0.91 (2)	C32—H32	0.9500
N1—C5	1.3711 (12)	C33—C34	1.3937 (15)
N1—N2	1.3711 (11)	C33—H33	0.9500
N1—C11	1.4341 (13)	C34—C35	1.4006 (15)
N2—C3	1.3457 (13)	C35—C36	1.3833 (15)
C3—C4	1.4131 (14)	C35—H35	0.9500
C3—C31	1.4798 (14)	C36—H36	0.9500
C4—C5	1.3790 (14)	C37—H37A	0.9800
C4—H4	0.9500	C37—H37B	0.9800
C5—C51	1.4827 (13)	C37—H37C	0.9800
C11—C12	1.3904 (14)	C51—C56	1.4041 (14)
C11—C16	1.3912 (14)	C51—C52	1.4063 (14)
C12—C13	1.3945 (15)	C52—C53	1.4025 (14)
C12—H12	0.9500	C53—C54	1.3881 (17)
C13—C14	1.3863 (18)	C53—H53	0.9500
C13—H13	0.9500	C54—C55	1.3940 (16)
C14—C15	1.387 (2)	C54—H54	0.9500
C14—H14	0.9500	C55—C56	1.3873 (14)
C15—C16	1.3924 (17)	C55—H55	0.9500
C15—H15	0.9500	C56—H56	0.9500
C16—H16	0.9500		
			
C34—O1—C37	118.12 (9)	C33—C32—H32	119.0
C52—O2—H2	110.0 (12)	C34—C33—C32	119.22 (10)
C5—N1—N2	111.75 (8)	C34—C33—H33	120.4
C5—N1—C11	129.15 (8)	C32—C33—H33	120.4
N2—N1—C11	119.09 (8)	O1—C34—C33	124.86 (10)
C3—N2—N1	105.23 (8)	O1—C34—C35	115.55 (9)
N2—C3—C4	110.50 (9)	C33—C34—C35	119.57 (9)
N2—C3—C31	121.73 (9)	C36—C35—C34	120.59 (10)
C4—C3—C31	127.77 (9)	C36—C35—H35	119.7
C5—C4—C3	106.20 (9)	C34—C35—H35	119.7
C5—C4—H4	126.9	C35—C36—C31	120.92 (10)
C3—C4—H4	126.9	C35—C36—H36	119.5
N1—C5—C4	106.31 (8)	C31—C36—H36	119.5
N1—C5—C51	123.26 (9)	O1—C37—H37A	109.5
C4—C5—C51	130.28 (9)	O1—C37—H37B	109.5
C12—C11—C16	120.92 (10)	H37A—C37—H37B	109.5
C12—C11—N1	119.50 (9)	O1—C37—H37C	109.5
C16—C11—N1	119.58 (9)	H37A—C37—H37C	109.5
C11—C12—C13	119.27 (10)	H37B—C37—H37C	109.5
C11—C12—H12	120.4	C56—C51—C52	118.79 (9)
C13—C12—H12	120.4	C56—C51—C5	120.96 (9)
C14—C13—C12	120.18 (11)	C52—C51—C5	120.18 (9)
C14—C13—H13	119.9	O2—C52—C53	121.86 (9)
C12—C13—H13	119.9	O2—C52—C51	118.34 (9)
C13—C14—C15	120.09 (11)	C53—C52—C51	119.79 (10)
C13—C14—H14	120.0	C54—C53—C52	120.35 (10)
C15—C14—H14	120.0	C54—C53—H53	119.8
C14—C15—C16	120.45 (11)	C52—C53—H53	119.8
C14—C15—H15	119.8	C53—C54—C55	120.25 (10)
C16—C15—H15	119.8	C53—C54—H54	119.9
C11—C16—C15	119.08 (11)	C55—C54—H54	119.9
C11—C16—H16	120.5	C56—C55—C54	119.64 (10)
C15—C16—H16	120.5	C56—C55—H55	120.2
C32—C31—C36	117.71 (9)	C54—C55—H55	120.2
C32—C31—C3	120.64 (9)	C55—C56—C51	121.13 (10)
C36—C31—C3	121.64 (9)	C55—C56—H56	119.4
C31—C32—C33	121.98 (10)	C51—C56—H56	119.4
C31—C32—H32	119.0		
			
C5—N1—N2—C3	−0.35 (11)	C36—C31—C32—C33	−0.50 (16)
C11—N1—N2—C3	178.79 (8)	C3—C31—C32—C33	−179.54 (10)
N1—N2—C3—C4	−0.10 (11)	C31—C32—C33—C34	−0.12 (17)
N1—N2—C3—C31	179.72 (9)	C37—O1—C34—C33	−4.99 (16)
N2—C3—C4—C5	0.51 (12)	C37—O1—C34—C35	176.10 (11)
C31—C3—C4—C5	−179.30 (10)	C32—C33—C34—O1	−178.32 (10)
N2—N1—C5—C4	0.67 (11)	C32—C33—C34—C35	0.54 (17)
C11—N1—C5—C4	−178.37 (9)	O1—C34—C35—C36	178.63 (11)
N2—N1—C5—C51	−175.35 (9)	C33—C34—C35—C36	−0.33 (17)
C11—N1—C5—C51	5.61 (16)	C34—C35—C36—C31	−0.31 (18)
C3—C4—C5—N1	−0.69 (11)	C32—C31—C36—C35	0.72 (17)
C3—C4—C5—C51	174.95 (10)	C3—C31—C36—C35	179.74 (10)
C5—N1—C11—C12	−131.50 (11)	N1—C5—C51—C56	50.34 (14)
N2—N1—C11—C12	49.52 (13)	C4—C5—C51—C56	−124.66 (12)
C5—N1—C11—C16	48.36 (15)	N1—C5—C51—C52	−132.78 (10)
N2—N1—C11—C16	−130.62 (10)	C4—C5—C51—C52	52.22 (15)
C16—C11—C12—C13	−0.83 (16)	C56—C51—C52—O2	179.78 (9)
N1—C11—C12—C13	179.03 (9)	C5—C51—C52—O2	2.83 (14)
C11—C12—C13—C14	0.03 (17)	C56—C51—C52—C53	−1.56 (15)
C12—C13—C14—C15	0.71 (19)	C5—C51—C52—C53	−178.50 (9)
C13—C14—C15—C16	−0.7 (2)	O2—C52—C53—C54	−179.36 (10)
C12—C11—C16—C15	0.87 (17)	C51—C52—C53—C54	2.03 (16)
N1—C11—C16—C15	−178.98 (10)	C52—C53—C54—C55	−0.71 (17)
C14—C15—C16—C11	−0.13 (19)	C53—C54—C55—C56	−1.06 (17)
N2—C3—C31—C32	−163.44 (10)	C54—C55—C56—C51	1.52 (16)
C4—C3—C31—C32	16.36 (16)	C52—C51—C56—C55	−0.20 (15)
N2—C3—C31—C36	17.57 (15)	C5—C51—C56—C55	176.71 (10)
C4—C3—C31—C36	−162.64 (11)		

### Hydrogen-bond geometry (Å, °)

**Table:**

*D*—H···*A*	*D*—H	H···*A*	*D*···*A*	*D*—H···*A*
O2—H2···N2^i^	0.91 (2)	1.88 (2)	2.7894 (11)	175.0 (17)

Symmetry codes: (i) *x*, −*y*+3/2, *z*−1/2.
